# Non-local Optical Topological Transitions and Critical States in Electromagnetic Metamaterials

**DOI:** 10.1038/srep17824

**Published:** 2015-12-16

**Authors:** Satoshi Ishii, Evgenii Narimanov

**Affiliations:** 1 Birck Nanotechnology Center and School of Electrical and Computer Engineering, Purdue University, West Lafayette, IN 47907, USA; 2 International Center for Materials Nanoarchitectonics (MANA), National Institute for Materials Science (NIMS), Tsukuba, Ibaraki 305-0044, Japan

## Abstract

Just as the topology of the Fermi surface defines the properties of the free electrons in metals and semiconductors, the geometry of the iso-frequency surface in the phase space of the propagating electromagnetic waves, determines the optical properties of the corresponding optical materials. Furthermore, in the direct analog to the Lifshitz transition in condensed matter physics, a change in the topology of iso-frequency surface has a dramatic effect on the emission, propagation and scattering of the electromagnetic waves. Here, we uncover a new class of such optical topological transitions in metamaterials, induced by the non-locality of the electromagnetic response inherent to these composites.

With the underlying quantum-mechanical wave nature of free electrons in solids, many optical phenomena find their close analogues in condensed matter physics - from optical solitons^1^ vs. charge density waves^2^ to Bose-Einstein Condensation^3^ and its optical equivalent in photorefractive crystals^4^. On a more basic level, both the “electronic” and “electromagnetic” material properties can be traced to the geometry of the underlying surface that constrains the phase space that is allowed to the propagating light or matter waves for a given energy. In condensed matter physics, this is the familiar Fermi surface – and change its topology from a “closed” to an “open” form in what is known as the Lifshitz transition^5^, leads to a profound effect on the transport properties of metals and semiconductors. First studied in metals, Lifshitz transitions have been observed in other materials such as superconductors^6^ and bilayer graphene^7^, proven to be general phenomena in condensed matters. The corresponding optical topological transitions^8^ (OTTs) that were recently discovered in electromagnetic metamaterials, show even more dramatic manifestations, as these optical effects are not subject to the restrictions of Fermi exclusion principle. A closed iso-frequency surface corresponds to a dielectric, and reduces to a simple sphere when the material does not show any anisotropy in its electromagnetic response. An open iso-frequency surface, on the other hand, is the key property of the hyperbolic media, where the wavenumbers of the propagating waves are not limited by the frequency, essentially breaking the conventional diffraction limit and leading to a broad bandwidth singularity of the photonic density of states^9^ in the effective medium limit. It is this key feature of the hyperbolic metamaterials (HMMs) that lead to the recent progress in super-resolution hyperlens imaging^10^,^11^,^12^,^13^, diffraction-unlimited photolithography and sub-wavelength interference^14^,^15^. A change in the topology of the iso-frequency surface therefore has a dramatic effect on a wide range of optical phenomena.

In its simplest form a transition in the topology of the iso-frequency surface can be observed in strongly anisotropic materials at the frequency when one of the dielectric permittivity components changes sign, and the iso-frequency surface transforms from a (closed) ellipsoid into an (open) hyperboloid^8^. Naturally, being induced by the changes of the average electromagnetic response, such a transition can be understood in the effective medium limit, which ignores the finite size of the unit cell of the material. However, as we show in the present work, the electromagnetic response of a metamaterial composite that results from a finite size of the unit cell, generally referred to as the non-locality or the spatial dispersion– can also lead to a different class of OTTs that has no counterpart in the effective medium approximation.

Aside from the direct consequences of this behavior for optical response of such media and the corresponding practical applications (in e.g. optical filters, electromagnetic modulators, etc.), this new class of OTTs introduced in the present work, has broader implications. As OTTs in electromagnetic metamaterials can be mapped onto the metric signature transitions in cosmology, the electromagnetic response of a non-local metamaterial can be connected to space-time discretization models^16^,^17^, with the metamaterial unit cell playing the role of the effective Planck length.

A hyperbolic iso-frequency surface for propagating electromagnetic waves generally implies that either one or two components of the dielectric permittivity tensor of the medium are negative. If the medium has an axis of symmetry (which is the case of most hyperbolic media studied so far^18^), in the effective medium limits the resulting dispersion equation for the TM-polarized waves can be expresses as,


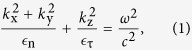


where *z* is the symmetry axis of the uniaxial material, *k* = (*k*_x_, *k*_y_, *k*_z_) is the wavevector, *ε* = diag(*ε*_τ_, *ε*_τ_, *ε*_n_) is the corresponding dielectric permittivity tensor, and *ω* is the electromagnetic wave frequency. Note that, depending on whether only one *ε*_z_ = *ε*_n_ or two components *ε*_x_ = *ε*_y_ = *ε*_τ_ of the dielectric permittivity tensor are negative, the hyperbolic iso-frequency surface corresponding to the equation (1), consists respectively of two separate parts (Fig. 1a) or a single sheet (Fig. 1b).

Note that these two different types of hyperbolic iso-frequency surface correspond to fundamentally different topological phases. In one case (see Fig. 1a), the iso-surface consists of two parts with each being a simply-connected topological space, while the other (Fig. 1b) is not simply-connected. The topological concept of simple connectivity (or equivalently 1-connectivity)^19^ describes the situation when every path between two points of a topological space can be continuously transformed, staying within the space, into any other such path while preserving the two endpoints. Alternatively, simple connectivity means that every loop on the surface can be contracted to a point. The continuous transformation of the orange loop in Fig. 1 illustrates the simple connectedness in the bottom surface in Fig. 1a and the lack of it in Fig. 1b. Note that this topological difference between the two hyperbolic iso-frequency surfaces also has profound physics implications. The lack of simple-connectedness indicates the presence of the holes in the surface - which implies the absence of the propagating waves in the corresponding range. For light incident on such material, this would imply a total (or nearly total, if the material losses are taken into account) reflection. Indeed, the reflectivity calculations of Figs. 1d,e for the two topologically different hyperbolic phases (corresponding to light incident onto sapphire, respectively in the 2nd and 3rd hyperbolic bands of sapphire - see Fig. 1c) show a qualitatively different reflectivity pattern.

In the strongly anisotropic regime the electromagnetic response of a material generally shows strong frequency variation, *ε* = *ε*(*ω*), leading to the hyperbolic dispersion in a limited wavelength range. This behavior is illustrated in Fig. 1c, using the example of sapphire that supports three different hyperbolic intervals (“red” and “green” regions in panel Fig. 1c). These hyperbolic bands are generally terminated at the frequencies when one of the dielectric permittivity components changes sign. As in such strongly anisotropic materials, this may not happen simultaneously with all eigenvalues of *ε*), a hyperbolic band is usually bounded by an “elliptical” phase (when all permittivity components are positive, “blue” regions in Fig. 1c), corresponding to a dielectric behavior, or a “metallic” one (*ε* < 0). Other natural hyperbolic materials (such as e.g. bismuth^20^, boron nitride^21^, and graphite^22^) also show a similar pattern.

Metamaterials - artificial electromagnetic composites patterned on a subwavelength scale that allow to “design” the unit cell for the desired electromagnetic response − also show the hyperbolic subbands and the corresponding topological transitions at their limits. This is illustrated in Fig. 2b–c, where we show the entire “phase diagram” for planar metamaterials. If the periodicity (*h*) of multilayers in Fig. 2a forming the planar metamaterial is sufficiently small compared to the free space wavenumber (*k*_0_) 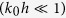, the dielectric permittivity tensor of the composite can be calculated using by the effective medium theory (EMT)^23^ which yields,


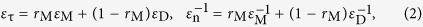


where *ε*_M_ and *ε*_D_ are the permittivities of the metal and the dielectric, respectively, *r*_M_ is the metal filling ratio and the x-y plane is parallel to the layers. For the TE-polarized waves, the corresponding effective medium dispersion is,


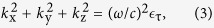


corresponding to either the spherical iso-frequency surface (for *ε*_τ_ > 0) or metallic behavior (*ε*_τ_ < 0) when the material does not support any TE propagating waves - see Fig. 2b. The TM-polarized modes, on the other hand, have a more complicated behavior as shown in Fig. 2c. With the dispersion defined by equation (1), the resulting phase diagram shows the metallic, the dielectric, and both kinds of the hyperbolic phases, depending on the frequency (the vertical axis of Figs. 2b,c) and the unit cell composition (horizontal axis of Fig. 2b,c). Note that, similar to the case of natural hyperbolic media, for any volume fraction *r* ≠ 0.5, the hyperbolic range is bounded by either a “metallic” or a “dielectric” phase.

Quantitatively, OTTs, which is the optical analog of Lifshitz transition in condensed mater physics, can be characterized by the topological phase parameter *p* defined as,


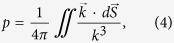


where *S* is area. As can be seen from equation (4), the topological phase parameter *p* is essentially the scaled solid angle in the phase space that is covered by the iso-frequency surface. This topological phase parameter is equal to the unity for a closed iso-frequency surface of a dielectric, is exactly zero for a metal, and lies between these two values for a hyperbolic medium. In Fig. 3, we plot the order parameter defined by equation (4) for the TM modes in planar metamaterials. Note a clearly visible difference in the values of the parameter *p* for the four phases (see Fig. 2c) supported by the system.

Using the example of a planar metamaterial (Fig. 2a) formed by the alternating layers of a dielectric with the permittivity *ε*_D_ = 4 and a Drude metal with the permittivity 
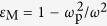
 and the plasma frequency *ω*_p_, in Fig. 4 we use the effective medium approximation (eq. (2)) to plot dispersion diagram *ω*(*k*_τ_, *k*_n_), where *k*_τ_ and *k*_n_ are the wavevector components in respectively the directions parallel and perpendicular to the layers. There, Fig. 4b corresponds to the metal volume fraction *r*_M_ = 1/3 < 0.5, with a dielectric phase between two hyperbolic bands, while Fig. 4c shows the dispersion diagram for *r*_M_ = 2/3 > 0.5, when a frequency bandgap (corresponding to the metallic phase) separates the topologically different hyperbolic phases. There, the geometry of the corresponding iso-frequency surface relates to the cross-section of the dispersion diagram in Fig. 4a at the given frequency *ω*.

Note that neither for *r*_M_ < 0.5 nor when *r*_M_ > 0.5 does the effective medium result of Fig. 4 allow for a “direct” topological transition between the hyperbolic phases of different topology. Instead, the corresponding evolution involves an intermediate phase, which can be either dielectric (*r*_M_ < 0.5) or metallic (*r*_M_ > 0.5).

However, the effective medium approximation (eq. (2)) used to calculate the phase space (Fig. 3) and dispersion (Fig. 4) diagrams for planar metamaterials, does not take into account the finite size of the metamaterial unit cell and the corresponding non-locality. While the resulting “spatial dispersion” effects (As the effects of the finite size of the unit cell can be incorporated into the dielectric permittivity tensor formalism via the *k*-dependence of *ε*, the corresponding mathematical description in terms of the “spatial frequency” *k* carries an essential similarity to that of the “temporal” frequency dispersion *ε*(*ω*). The term “spatial dispersion” originates from this mathematical connection.) are generally weak, especially for increasingly smaller size of the (subwavelength) unit cell, they may account for qualitatively new physics such as e.g. formation of additional waves^23^,^24^,^25^,^26^,^27^,^28^,^29^,^30^, double-refraction^31^,^32^, and may therefore have the potential to lead to an entire new class of OTTs, not found within the conventional effective media. In particular, we predict that essential non-locality of optical metamaterials leads to the direct transitions between two topologically different hyperbolic phases.

Indeed, the exact dispersion diagram for metal-dielectric composite of Fig. 2a does uncover the predicted topological transition. Solving for the exact propagating wave dispersion^33^ (see Methods) in the metal-dielectric composite having the identical material properties and geometries as before, we obtain the diagram shown in Fig. 5 at *r*_M_ = 2/3. As we increase the frequency, at the critical point (*ω*_c_) the originally single-sheet connected hyperboloid (Fig. 5c) degenerates into a conical surface (Fig. 5b) which then immediately evolves into the disconnected hyperboloids of Fig. 5a. This behavior is further illustrated in the constant frequency cross-sections (see Fig. 5d–f) and the full dispersion diagram of Fig. 5g plotted in *k*_τ_-*k*_n_-*ω* space.

Note that the conical dispersion surface at the OTT allows an arbitrarily small wavenumber including *k* = 0 to propagate in multilayer metamaterials. This formally allows for zero effective refractive index at the transition, thus extending the original concept of epsilon-near-zero materials^34^ to an entirely new class of optical modes.

Furthermore, the conical iso-frequency surface at the non-local OTT predicted in the present work, will result in the phenomenon of conical diffraction^35^ that manifests in the “double-refraction” of a TM-polarized beam at normal incidence upon the planar metamaterial surface. It should be straightforward to observe this effect in the standard experimental setup for optical refraction in planar metamaterials^36^.

In the present work, we focus on the fundamental concept and the general properties of non-local optical topological transitions. As the iso-frequency surface and its topology is a fundamental property of propagating electromagnetic waves in a medium and is directly related to the photonic density of states, such transitions will have a strong effect on a broad range of optical phenomena - from light localization and scattering to nonlinear and quantum optics. Furthermore, multilayer hyperbolic metamaterials such as the planar composite studied in the present work, facilitated by the immediate availability of the material components (e.g. transparent conducting oxides in near infrared^37^ and heavily-doped semiconductors mid infrared^36^) and well-established fabrication protocols (physical vapor deposition techniques such as molecular beam epitaxy and sputtering that enable multilayers with sub-nanometer accuracy), show immediate promise as spatial optical filters, and further research is expected leading to many other applications. To further tailor the applicability of such structures, passive dielectric layers can be replaced with active dielectric layers for loss compensation^38^,^39^.

To summarize, we present that non-locality can induce a new type of OTT in electromagnetic metamaterials, with the system at the critical point corresponding to a new class of zero-index media.

## Methods

Wave propagation through multilayers consisting of isotropic materials can be described by the standard transmission matrix approach^33^. For the case with periodic bilayers, the final dispersion equations for TE- and TM-polarized waves reduce to,


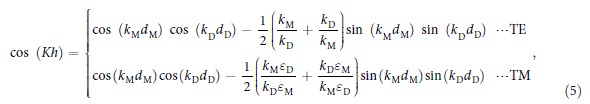


where *d*_M_ (=*hr*_M_) and *d*_D_ (=*h*(1 − *r*_M_)) are the thicknesses of the metal layer and the dielectric layer, respectively, *K* is the wavenumber for Bloch waves, 
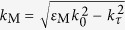
 and 
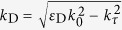
, and *k*_τ_ is the wavevector component parallel to the layers. The effective medium approximation of equation (2) is based on the second-order expansion of equation (5) in powers of *kd* ≪1.

**Figure 4:**
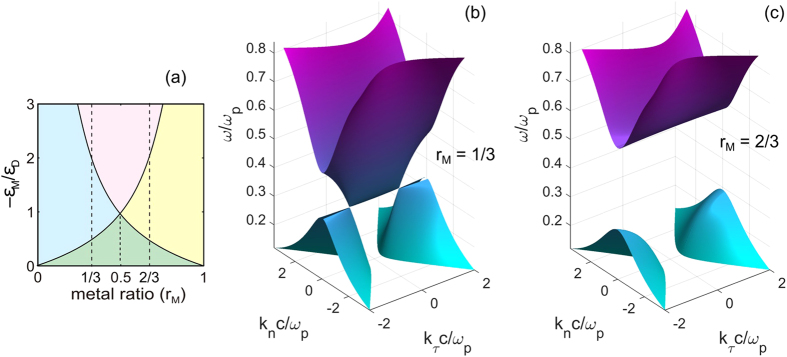
Optical topological transitions in multilayer HMM for TM polarization. (**a**) The topology map of iso-frequency surface of TM-polarized waves supported by a multilayer HMM. The two dashed lines are at *r*_M_ = 1/3 and *r*_M_ = 2/3. (**b**,**c**), Optical topological transitions at *r*_M_ = 1/3 (**b**) and *r*_M_ = 2/3 (**c**).

**Figure 5:**
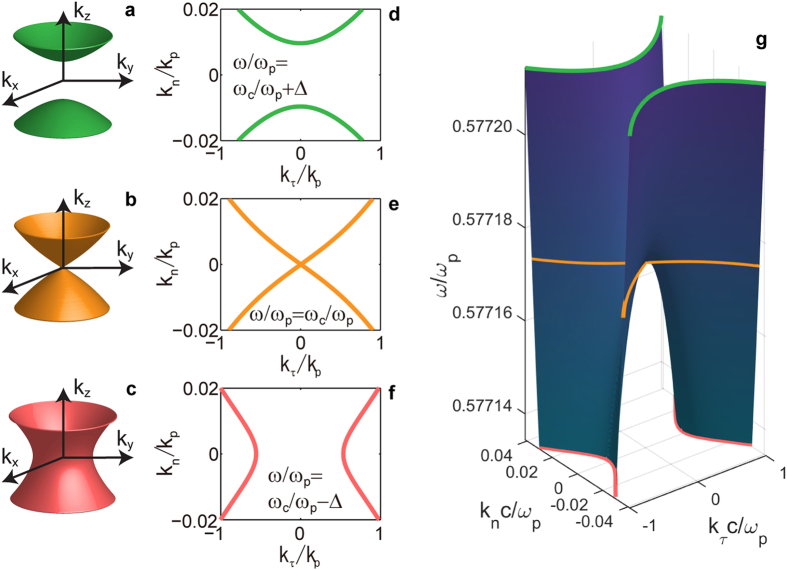
Three classes of propagating wave dispersion in multilayer HMM. (**a**–**f**) Iso-frequency curves of the multilayer HMM in k_x_-k_y_-k_z_ space (**a–c**) and in k_τ_-k_n_ space **(d–f**) at *ω*/*ω*_p_ = *ω*_c_/*ω*_p_+Δ (**a**,**d**), *ω*/*ω*_p_ = *ω*_c_/*ω*_p_ (**b**,**e**) and *ω*/*ω*_p_ = *ω*_c_/*ω*_p_−Δ (**c**,**f**). Panel (**g)** shows the evolution of iso-frequency curve with frequency, in the range *ω*_c_/*ω*_p_ − Δ ≤ *ω*/*ω*_p_ ≤ *ω*_c_/*ω*_p_ + Δ. The iso-frequency curves in panel (**d**–**f**) are superimposed in panel (**g**) with the same colors. In the entire plot, *r*_M_ = 2/3 and Δ = 2.5 × 10^−5^.

For the dispersion calculation shown in Fig. 5, loss-less Drude model was used for the metal and the permittivity of the dielectric was set to *ε*_D_ = 4.0 same as in Fig. 4. The plasma frequency was defined as a normalized parameter *ω*_p_ = 2*πc*/40*h* (*c* is the speed of light) using the period (*h*), thus we can work in the metamaterial limit (*k*_0_*h* ≪ 1) at around the surface plasmon resonance frequency (*ω* ≈ 2*πc*/90*h*). When *h* ≈ 30 nm, the value of *ω*_p_ (≈0.5 eV) corresponds to a typical plasma frequency of a highly-doped transparent conductive oxides which is one of the alternative plasmonic materials studied extensively in the past few years (see Ref. 37). Also note that the chosen value *ε*_D_ = 4.0 is consistent with the dielectric permittivities of a number of transparent oxides such as zinc oxide and titanium oxide.

## Additional Information

**How to cite this article**: Ishii, S. and Narimanov, E. Non-local Optical Topological Transitions and Critical States in Electromagnetic Metamaterials. *Sci. Rep.* **5**, 17824; doi: 10.1038/srep17824 (2015).

## Figures and Tables

**Figure 1:**
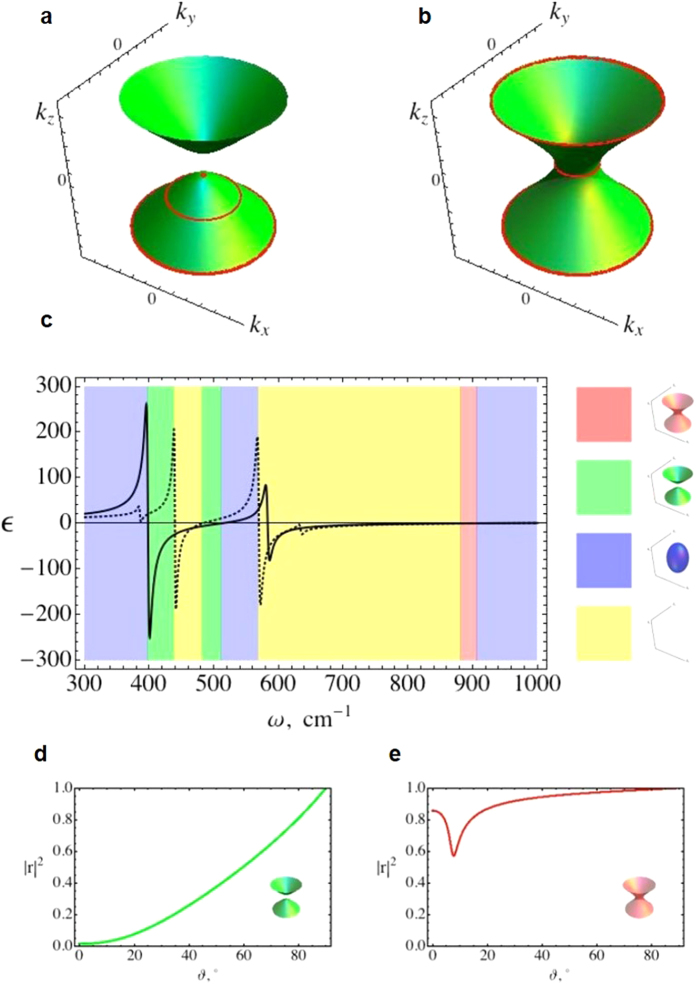
Iso-frequency surfaces of hyperbolic metamaterials and frequency-dependent anisotropic permittivity and reflectance of sapphire. (**a**,**b**) Iso-frequency surfaces of hyperbolic metamaterials, for Re[ε_τ_] > 0, Re[ε_n_] < 0 (**a**) and Re[ε_τ_] < 0, Re[ε_n_] > 0 (**b**). Each of the two sheets forming the iso-frequency surface in panel a, is simply connected, as any closed loop it contains, can be continuously transformed into a single point. Note that this is not the case in panel (**b**). (**c**) Frequency-dependent anisotropic permittivity of sapphire, from the date of ref. 40. The solid and dashed lines show ordinarily and extraordinarily components, respectively. (**d**–**e**), Angular dependence of sapphire reflectivity of sapphire in a hyperbolic frequency bands, when iso-frequency surface is (**d**) and is not (**e**) simply-connected. The color code (green and red) in panel (**c**) corresponds to the colors in panels (**d**) and (**e**).

**Figure 2:**
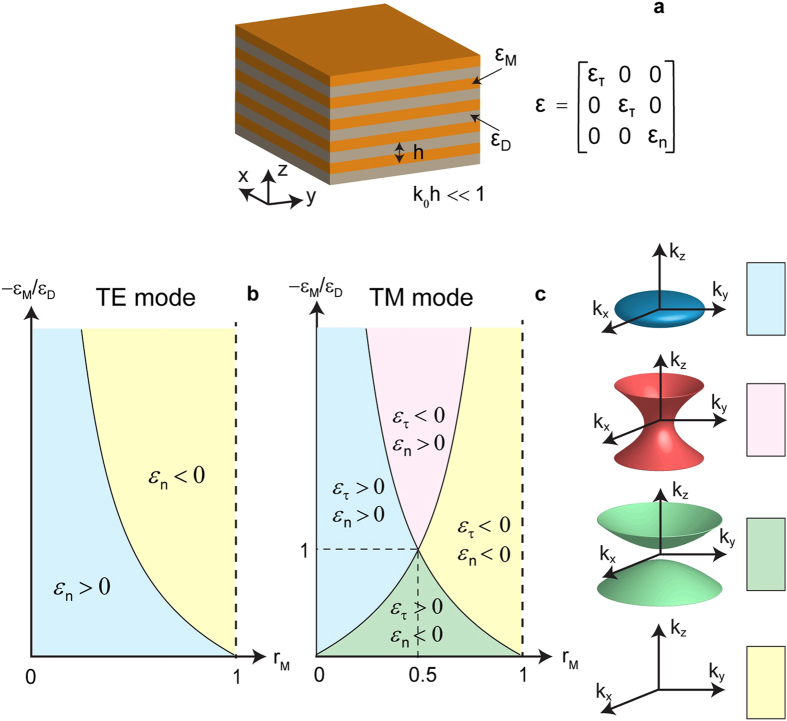
The topology of iso-frequency surface of a multilayer HMM. (**a**) Schematic of a multilayer HMM. (**b**–**c**) The topology map of iso-frequency surfaces in TE (**b**) and TM (**c**) polarizations. Right side shows the correspondence of the iso-frequency surfaces to their color.

**Figure 3:**
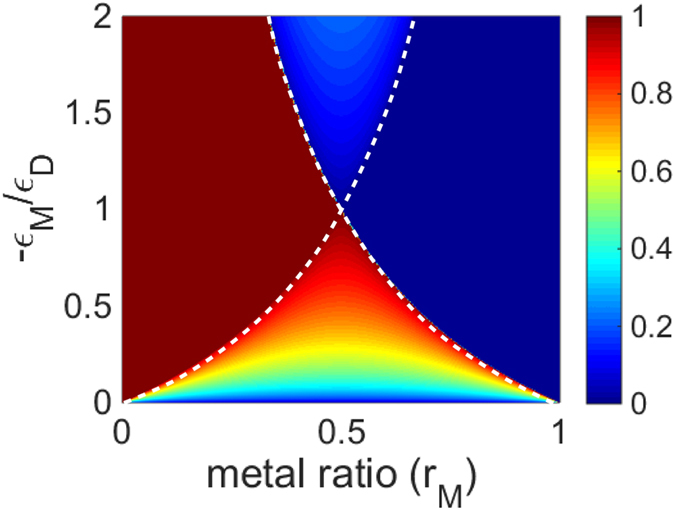
Topological phase parameter of the multilayer HMM for TM polarization. The false-color scale representation of the topological phase parameter (*p*) in the multilayer hyperbolic metamaterial as a function of the metal volume fraction (horizontal axis) and the ratio of the metal and dielectric components permittivities (vertical axis), for TM-polarized propagating waves.
